# Dedifferentiation process driven by radiotherapy-induced HMGB1/TLR2/YAP/HIF-1α signaling enhances pancreatic cancer stemness

**DOI:** 10.1038/s41419-019-1956-8

**Published:** 2019-09-26

**Authors:** Lirong Zhang, Hui Shi, Hongbo Chen, Aihua Gong, Yanfang Liu, Lian Song, Xuewen Xu, Tao You, Xin Fan, Dongqing Wang, Fang Cheng, Haitao Zhu

**Affiliations:** 1 The Affiliated Hospital of Jiangsu University, 212001 Zhenjiang, China; 2 School of Pharmaceutical Sciences (Shenzhen), SYSU, 518107 Shenzhen, China; 3 School of Medicine, Jiangsu University, 212013 Zhenjiang, China; 4 The First People’s Hospital of Zhenjiang, 212001 Zhenjiang, China; 5 Faculty of Science and Engineering, ÅboAkademi University and Turku Centre for Biotechnology, FI-20520 Turku, Finland

**Keywords:** Radiotherapy, Cell death

## Abstract

Differentiated cancer cells reacquiring stem cell traits following radiotherapy may enrich cancer stem cells and accelerate tumor recurrence and metastasis. We are interested in the mechanistic role of dying cells-derived HMGB1 in CD133^−^ pancreatic cancer cells dedifferentiation following radiotherapy. We firstly confirmed that X-ray irradiation induced differentiation of CD133^−^ pancreatic cancer cells, from either sorted from patient samples or established cell lines, into cancer stem-like cells (iCSCs). Using an in vitro coculture model, X-ray irradiation induced dying cells to release HMGB1, which further promoted CD133^−^ pancreatic cancer cells regaining stem cell traits, such as higher sphere forming ability and expressed higher level of stemness-related genes and proteins. Inhibiting the expression and activity of HMGB1 attenuated the dedifferentiation stimulating effect of irradiated, dying cells on C133^−^ pancreatic cancer cells in vitro and in PDX models. Mechanistically, HMGB1 binding with TLR2 receptor functions in a paracrine manner to affect CD133^−^ pancreatic cancer cells dedifferentiation via activating Hippo-YAP pathway and HIF-1α expression in oxygen independent manner in vitro and in vivo. We conclude that X-ray irradiation induces CD133^−^ pancreatic cancer cell dedifferentiation into a CSC phenotype, and inhibiting HMGB1 may be a strategy to prevent CSC enrichment and further pancreatic carcinoma relapse.

## Introduction

Pancreatic carcinoma (PC) constitutes the fourth leading cause of cancer mortality^1^. Systemic chemotherapy or radiotherapy is regarded as an international standard of care for advanced stage PC patients. However, more and more clinical evidence indicate that tumors develop adaptive response and become more aggressive following radiotherapy^2,3^. One of the most important mechanisms for resistance is the repopulation of cancer stem cells (CSCs)^4^.

CSCs are defined as tumor cells that express stem cell markers (cluster of differentiation 133, CD133), have the ability to self-renew and repopulate the whole tumor. Transcription factors (including Oct4, Sox2, Nanog, and c-Myc) are known to play crucial roles in maintaining the stemness and self-renewal abilities of cancer stem cells. CSCs play a pivotal role in tumor development, progression, and recurrence^5^. Current cancer treatments not only kill a bulk amount of tumor cells but cause the expansion of CSCs population. For decades, it had been believed that the expansion of CSCs ascribed to the accelerated repopulation CSCs following radiotherapy. Recently, more and more studies found that CSCs self-renewal and proliferation are unlikely to be the sole source of the enrichment of CSC numbers after irradiation^6,7^. It has been recently reported that differentiated non-cancer stem cells may also be able to reacquire stem cell traits under ionizing radiation stress in several solid tumors^8,9^. Lagadec et al. reconstituted a mixed population of breast cancer stem cells and non-cancer stem cells in vitro and confirmed that dedifferentiation of non-cancer stem cells was the main factor for CSCs enrichment^10^. It is still unknown whether the dedifferentiation of non-cancer stem cells also exists in PC following chemo- or radiotherapy.

Reexpression of pluripotency factors is the main mechanisms for radiotherapy induced dedifferentiation of cancer cells in the earlier stage studies. Multiple studies demonstrated that inflammatory factors (IL-6) can function in a paracrine or autocrine manner to affect the dedifferentiation of non-cancer stem cells^11,12^. Although tumor dedifferentiation is tightly regulated by genetic and epigenetic factors, exogenous signals from the tumor microenvironments also have a very important role in this process. Dying cells and their released damage associated molecular patterns (DAMPs) are predominant components of tumor microenvironment following radiotherapy. DAMP molecules such as calreticulin, adenosine triphosphate, or high-mobility group box 1 (HMGB1) bind to diverse surface receptors on the neighboring cancer cells and trigger further proliferation and metastasis. However, the molecular mechanism of DAMPs in cancer cells dedifferentiation following radiotherapy is still not clear and needs to be further investigated in detail.

HMGB1 is passively released from dying or injured cells or actively secreted from cancer cells in response to exogenous and endogenous stimuli such as hypoxia and various soluble factors (TNF-α and IL-1). HMGB1, released from tumor cells upon chemotherapy or radiotherapy, is an important component of disordered tumor microenvironment and highly associated with cancer hallmarks including sustain proliferation, resistance to cell death, invasion and metastasis, and recurrence^13–15^. Our previous work confirmed that dying cells derived HMGB1 accelerated pancreatic carcinoma metastasis following radiotherapy^16^. However, it is unclear whether HMGB1 secreted by dying cancer cells following radiotherapy is involved in expansion of CSCs, especially in the non-CSCs dedifferentiation. Therefore, we are interested if dying pancreatic cancer cells during radiotherapy activate HMGB1-mediated paracrine signaling events that promote dedifferentiation of resident non-CSCs.

To test this hypothesis, we used the Millicell coculture system and patient-derived xenograft (PDX) tumors to show that dying pancreatic cancer cells following radiotherapy significantly induced CD133^**−**^ cancer cells dedifferentiation in vitro and tumorigenesis in vivo. Silencing HMGB1 in irradiated cancer cells (feeder cells) attenuated expression levels of stem cell-related markers (Oct4, Sox2, C-myc, and Nanog) and sphere forming ability of CD133^**−**^ reporter cells. Finally, we showed that HMGB1 functions in a paracrine manner to activate Hippo-YAP pathway and enhances hypoxia-inducible factor 1α (HIF-1α) expression in CD133^−^ cells in an oxygen independent manner in vitro and in vivo.

## Materials and methods

### Cell culture and human pancreatic carcinoma primary specimens

Human pancreatic cancer cell line (PaTu8988) was obtained from Cell Bank of China Academy of Sciences (Shanghai, China). PaTu8988 cells, a human pancreatic carcinoma of ductal cell origin, were maintained in DMEM medium supplemented with 10% FBS, 100 U/mL penicillin, and 100 U/mL streptomycin. The cells were put in a humidified atmosphere of 95% air with 5% CO_2_ at 37 °C and passaged with 0.25% trypsin/EDTA every 3 days. Cells were passaged for <2 months after recovery.

Tumor specimens were obtained under a protocol approved by Department of General Surgery, the First Affiliated Hospital of Nanjing Medical University and the Affiliated Hospital of Jiangsu University. All patients underwent fully informed consent in accordance with local research ethics committee guidelines. Specimen site was selected to avoid both the tumor margin and necrotic core, and were kept overnight at 4 °C in DMEM with 10% FBS. Specimens were dissected into 1–2 mm^3^ cubes and digested in single-cell suspension and was filtered sequentially through 100 μm cell strainers, then centrifuged at 300 × *g* for 5 min, and washed in PBS. Human Tumor Dissociation Kit (Miltenyi Biotec.) was used to remove contaminating stromal cells for 2 h at 37 °C. The primary cancer cells were expanded for 1 weeks and then for further use.

### Irradiation and in vitro coculture system of cancer cells

Pancreatic cancer cells cultured in 6 cm dishes or Millcell insurts were irradiated at room temperature using an X-ray irradiator (Linear accelerator, Turebeam_STX, Varian, USA) with indicated dose (2, 4, 8, 10, and 20 Gy). The dose rate of the machine is about 4 Gy/min. Corresponding controls were sham irradiated. Irradiated cells were immediately trypsinized and reseeded for further use.

Segregated irradiated cancer cells and untreated cancer cells coculture system was established as previously reported^17^. In brief, 5 × 10^4^ irradiated indicated cancer cells were seeded on 0.4 μm inserts (Millicell) in DMEM with 2% FBS. After 12 h, the inserts were moved to 24-well plates containing indicated number untreated CD133^−^ cancer cells in DMEM with 2% FBS. Different concentration of recombinant human HMGB1 (rhHMGB1, 100, 200, 250, and 300 ng/mL) was added to the same medium above mentioned in the inserts as positive control. Empty inserts with the same medium were used as control. The experiments were repeated three times with duplicate samples per group.

### Flow cytometry and fluorescent-activated cell sorting

CD133 staining was carried out as described previously^18^. In brief, 5 × 10^6^ cells were harvested, disaggregated into a single-cell suspension, and incubated with 2 mg/ml mouse anti-human CD133/phytoerythrin (PE) antibody for 30 min at 4 °C in the dark. After incubation, the samples were washed with PBS and analyzed by FACS AriaII (Becton Dickinson, USA).

For separating CD133^+^ and CD133^−^ population by FACS, cultured pancreatic cancer cells growing in sphere forming media system (SFM, DMEM-F12 with 2%B27, 20 ng/ml epidermal growth factor (EGF), 20 ng/ml basic fibroblast growth factor (bFGF), 4 ug/ml heparin, and 5 μg/ml insulin, Sigma-Aldrich) were stained for CD133. Cancer cells were incubated with trypsin–EDTA, dissociated and passed through a 40 µm sieve. Cells were pelleted by centrifugation at 500 × *g* for 5 min at 4 °C, resuspended in 100 µL of monoclonal mouse anti-human CD133/PE antibody (1:10, Miltenyi Biotec.), and incubated for 30 min at 4 °C. The sorting gates were established using cells stained with isotype control PE-conjugated antibodies (BD pharmingen). Sorted CD133^+^ and CD133^−^ cells were reseeded for further use.

### Reagents treatment

Recombinant human (rhHMGB1, HMGBiotech, Germany) was dissolved in distilled water to make a 1000 ng/ml stock solution. When the cells grown to 80% confluency, various concentrations of rhHMGB1 (100, 200, 250, and 300 ng/mL) were added for the indicated time. The treated cells were subjected to the following experiments.

Ethyl pyruvate (EP, HMGB1 inhibitor) was purchased from MCE (USA). Cells were grown to 80% confluency, treated with EP (1:1000) for the indicated time, and subjected to the following experiments.

Stevioside (TLR2 antagonist) purchased from Topscience (Shanghai, China) and dissolved in dimethyl sulfoxide (DMSO). Cells were grown to 80% confluency, treated with 2 μM Stevioside for the indicated time, and subjected to the following experiments.

### In vitro sphere-forming assay

After sorted, CD133^−^ pancreatic cancer cells were seeded into ultra-low adhesion plates (Corning, NY, USA) and suspended in SFM system, ranging from 1 to 256 cells/well, for 1–2 weeks to allow formation of spheres from single cells. The culture medium was replaced by fresh medium every 2 days. After 1–2 weeks, the number and size of spheres in each well were quantified.

### RNAi and gene transfection

Pancreatic cancer cells were seeded in six-well plates at a density of 1 × 10^5^ cells/well to achieve a confluence of 70–80% overnight. Then, HMGB1-shRNA, TLR2-shRNA, YAP-shRNA, HIF-1α -shRNA, and negative control shRNA (Suzhou Ribo Life Science CO., Ltd, Suzhou, China) were transfected into cells, respectively, using transfection reagent (Lipofectamine 2000, Invitrogen, China) according to the manufacturer’s instructions. The specific shRNA sequences are listed in Supplementary Table 1.

For establishing the stable sh-HMGB1 cancer cells, the lentiviral packaging kit was purchased from Open GeneCopoeia. Lentivirus carrying HMGB1-shRNA1 and was packaged in 293T cells and concentrated from the supernatant, as instructed by the manufacturer's manual. Stable cell lines were established by infecting lentivirus into pancreatic cancer cells followed by puromycin (1 μg/ml) selection for 10–14 days. These established stable cell lines were maintained in DMEM containing 10% FBS and puromycin (0.75 μg/ml) for further experiments.

### Western blot analysis

Protein concentrations were determined by BCA method. Western blot assay was carried out as described previously^19^. In brief, after extraction, proteins in the cell lysate were resolved on 4–12% Criterion XTBis-Tris gels (Bio-Rad) and transferred to a nitrocellulose membrane. After blocking with 5% milk, membranes were incubated at 4 °C with various primary antibodies overnight. After incubation with peroxidase-conjugated secondary antibodies for 1 h at room temperature, the signals were visualized using enhanced or super chemiluminescence and exposure to X-ray films. Antibodies against HMGB1 and β-tubulin were purchased from Abcam Company (Cambridge, USA). Antibodies against CD133, Oct4, Sox2, Nanog, TLR2, TLR4, N-cadherin, Vimentin, E-cadherin, C-myc, p-MOB1, MOB1, YAP, p-YAP, HIF-1α, and Histone3 were obtained from Cell Signaling Technology, Inc. (Boston, USA). The secondary antibody preparations either anti-rabbit or anti-mouse were purchased from Boster Biotechnology Company (Wuhan, China).

Nuclear and cytoplasmic proteins were isolated with Nuclear and Cytoplasmic Extraction Kit (Thermo Scientific, Waltham, MA, USA). The protein from total cells was extracted from the cultured cells, which were homogenized in RIPA lysis buffer (1% NP40, 0.1% Sodium dodecyl sulfate (SDS), 100 μg/ml phenylmethylsulfonyl fluoride, 0.5% sodium deoxycholate, in PBS) on ice. The supernatants were collected after centrifugation at 12000 × *g* at 4 °C for 20 min. The separated proteins were then transferred to a PVDF membrane. The membrane blots were first probed with a primary antibody. β-tubulin was used as a loading control for cytosol protein or total protein, and Histone3 was used as a loading control for nuclear protein.

### Luciferase assay

CD133^−^ PaTu8988 cancer cells were transiently transfected with the YAP-Luciferase/Renilla (50:1) or HRE/Renilla(50:1) reporter plasmid using transfection reagent (Lipofectamine 2000, Invitrogen, Mississauga, Ontario, Canada) according to the manufacturer’s instructions. Luciferase assay was performed by using the Dual Luciferase Assay System kit after transfection. Activity was assayed in three independent experiments.

### Quantitative real-time PCR

Total RNA was extracted from cancer cells using the RNeasy kit (QIAGEN). For mRNA analysis, cDNA was synthesized from 1 μg total RNA using the Superscript III kit (Invitrogen). SYBR Green-based real-time PCR was subsequently performed in triplicate using SYBRGreen master mix (SA Biosciences) on an Applied Biosystems Step One Plus real-time PCR machine (Thermo Fisher Scientific). For analysis, the threshold cycle (Ct) values for each gene were normalized to expression levels of GAPDH. The primers used are listed in Supplementary Table 2.

### Chromatin immunoprecipitation (ChIP) assay

ChIP was performed according to the protocol of the chromatin immunoprecipitation assay kit. Briefly, cells (CD133^−^ PaTu8988 or HIF-1α knockdown CD133^−^ PaTu8988 cancer cells) were pretreated with or without rhHMGB1 under normoxia or hypoxia condition and then cross-linked in 3.7% formaldehyde for 15 min, quenched with glycine for 5 min, and lysed with SDS lysis buffer. Chromatin was sheared by sonication, and lysates were precleared with salmon sperm DNA/protein A agarose slurry for 1 h and incubated with IgG or antibodies against HIF-1α or YAP in the presence of protein A agarose beads overnight. After sequential washes of the agarose beads and eluted, the elutes were heated at 65 °C for 4 h to reverse the cross-linking and treated with RNase A for 30 min at 37 °C, followed by treatment with proteinase K for 1 h at 45 °C to remove RNA and protein. DNA was recovered, eluted, and then assayed using PCR. The primer for the human Oct4, SOX2, C-myc, and Nanog promoter containing the HRE or TEAD site listed in Supplementary Tables 3 and 4, respectively.

### Co-immunoprecipitation assay

Cells were collected and lysed in 0.5 ml lysis buffer plus protease inhibitors for 50 min on a rotor at 4 °C. After 12,000 × *g* centrifugation for 15 min, the lysates were immunoprecipitated with 2 μg specific antibody overnight at 4 °C, and 30 μl A/G agarose beads (Santa Cruz, SC-2003) were washed and then added for an additional 6 h. Thereafter, the precipitants were washed three times with lysis buffer, and the immune complexes were boiled with loading buffer for 5 min and analyzed by SDS–PAGE. The following antibodies were used for immunoprecipitation: antibodies against normal Rabbit IgG [2729, CST], anti-TLR2 [D7G9Z, CST], and Anti-YAP [14074, CST].

### Xenograft tumor models

Animal studies were approved by the Committee on the Use of Live Animals for Teaching and Research of the Jiangsu University. Female BALB/c nude mice (purchased from The Compare Medicine Center, Yangzhou University, China), ages 4 weeks, were maintained under standard conditions according to institutional guidelines.

Sorted CD133^−^ PaTu8988 cancer cells were cocultured with irradiated parental cancer cells HMGB1^+^ cells(HMGB1 positive, HP), irradiated HMGB1 knock down pancreatic cancer cells (iHMGB1shRNA1), rhHMGB1(150 ng/mL) and the same empty medium (PBS). Fourteen days later, 1 × 10^3^, 1 × 10^4^ and 1 × 10^5^ cells/mouse were injected into the subcutaneous of nude mice, respectively. Mice were monitored every day until the end point of day 50, when the tumors that were palpable were taken as a positive.

1 × 10^6^ CD133^−^ PaTu8988 cancer cells, TLR2^−^CD133^−^ PaTu8988 (TLR2 knockdown CD133^−^ PaTu8988), YAP^−^CD133^−^ PaTu8988 (YAP knockdown CD133^−^ PaTu8988), or HIF-1α^−^ CD133^−^ PaTu8988 (HIF-1α knockdown CD133^−^ PaTu8988) cancer cells were implanted subcutaneously into the right dorsal flanks of nude mice, respectively. When the tumors reached a volume of 200 mm^3^, the mice were treated with HMGB1 by peritumoral injection every 2 days for 2 weeks. Tumors were harvested 3 days after the last injection for analysis of CD133^+^ cells, and expression of stem cell-related markers (Nanog and Oct4).

1 × 10^6^ PaTu8988 cells were implanted into the nude mice. When the tumor reached a volume of 200 mm^3^, the mice were received 20 Gy X-ray irradiation. Two days later, the mice were randomized to receive surrounding tumor injections of PBS, EP (HMGB1 inhibitor), Stevioside (TLR2 inhibitor), Verteporfin(YAP inhibitor), or LW6 (HIF-1α inhibitor) for 5 days (designed for day1). The experiment was terminated on 30 day. The tumor volume and regrowth speed were monitored.

### PDXs and in vivo experiments

NSG (NOD. Cg-*Prkdc*^*scid*^ *Il2*^*rgtm1Wjl*^/SzJ) mice were purchased from the BEIJING IDMO Co., Ltd. and maintained in Animal Center of Jiangsu University in compliance with the Guide for the Care and Use of Laboratory Animals (NIH Publication No. 85–23, revised 1996). The experimental protocols were approved by the Committee for Ethical Affairs of Jiangsu University (Zhenjiang, China), and the methods were carried out in accordance with the approved guidelines. All in vivo work was performed with a minimum of *n* = 3 mice per condition.

Serial passaging of the PDX was carried out by implanting small fragments of the tumor subcutaneously into dorsal flanks of NSG mice. Experiments were performed using PDX tumors passages 4 and 5. HPCx1 and HPCx2 derived PDXs mice were fixed into special equipment and received 4Gy radiation for each 2 days and for five times. Seven days later, the mice were randomized to receive peritumoral injections of PBS, rhHMGB1 (150 ng/ml), and HMGB1 antibody (250 ng/ml) every 2 days for 2 weeks. The experiment was terminated on 30 day. Animal weight and tumor size were measured bi-dimensionally using calipers twice a week.

### Immunofluorescence analyses

Cells were seeded on coverslips. Cells were fixed with 4% paraformaldehyde for 10 min at room temperature, and then permeabilized with 0.1% Triton X-100. After blocking in goat serum for 1 h, slides were incubated with primary antibody (anti-YAP (1:100) and anti-HIF-1α (1:800)) for 1 h at 4 °C overnight. Slides washed three times with PBST and incubated with CY3-conjugated secondary antibodies (Invitrogen, 1:1000) for 1 h at room temperature. Slides washed three times with PBST and incubated with DAPI for 5 min at room temperature. The slides were then washed three times with PBST and mounted. Cell images were captured with a confocal microscope (Leica).

### Patient selection

The Cancer Genome Atlas (TCGA) database (https://tcga-data.nci.nih.gov/tcga; TCGA_PAAD_exp_HiSeqV2_percentile-2015-02-24TCGA pancreatic adenocarcinoma (PAAD) gene expression by RNAseq (IlluminaHiSeq percentile) including 183 pancreatic carcinoma patient specimens was utilized to further analyse the relationship between CD133, Oct4, Nanog, Sox2, YAP, and HIF-1α. High and low groups were defined as above and below the mean, respectively.

### Statistical analysis

All data are presented as the mean ± SEM (standard error of the mean). Linear regression and *F* test were used to determine the significance of TCGA data. The significances of differences between groups were analyzed using Student’s *t-*tests, one-way, or two-way ANOVA. Values of *P* < 0.05 were considered to be significant. All the experiments were repeated at least three times.

## Results

### X-ray irradiation induced cell death promotes CD133^−^ dedifferentiation

**Fig. 1:**
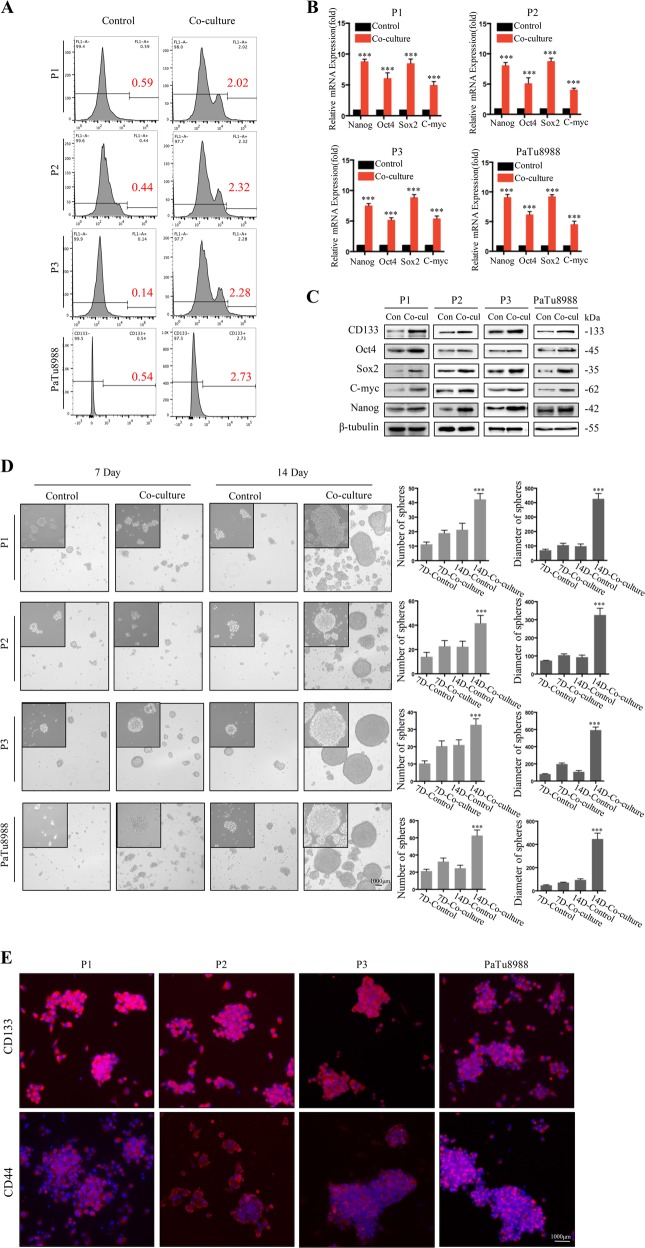
X-ray irradiation induced cell death promotes CD133^−^ dedifferentiation. Cocultured 20 Gy irradiated parental cancer cells (feeder cells) with sorted CD133^−^ cancer cells (reporter cells) for the indicated time. **a** Flow cytometry analysis the percentage of CD133^+^ cells in the reporter cell population following 7d co-culture. **b** qRT-PCR analysis of the gene expression level of stem cell-related markers (C-myc, Oct4, Sox2 and Nanog) in co-cultured CD133^−^ reporter cancer cell population following 7 days co-culture. **c** Western blot analyses the protein expression of stem cell-related markers (CD133, Oct4, Sox2, C-myc, and Nanog) in cocultured CD133^−^ reporter cell population following 7 days coculture. **d** Formation of sphere number and size was measured in cocultured CD133^−^ reporter cancer cell population following 7 days and 14 days coculture. **e** Immunofluorescence staining access the expression level of CD133 and CD44 in spheres from CD133^−^ reporter cancer cells following 14 days coculture. Experiments were repeated three times and the data were expressed as mean ± SEM. Student's *t*-test, oneway, two-sided ANOVA. *P < 0.05, **P < 0.01, ***P < 0.001

To test the hypothesis that irradiation-induced cell death promoted cancer cell dedifferentiation, we first confirmed that CD133^+^ cancer stem-like cells can be enriched by irradiation of primary pancreatic cancer cells from pancreatic carcinoma patients (P1, P2, and P3), or established pancreatic cancer cell line (PaTu8988) with clinically relevant doses in vitro (Fig. S1A). To test if these CSCs originated from dedifferentiation of CD133^−^ cancer cells, we cocultured lethally irradiated pancreatic cancer cells (20 Gy, lethal dose optimized in Fig. S1B) or nonirradiated control cells (0 Gy) together with sorted CD133^–^ cancer cells using a Millicell insert system. FACS analysis showed that compared with nonirradiated control cells, irradiated feeder cells led to more CD133^+^ cells dedifferentiated from CD133^−^ cancer cells following 7 days coculture (P1: 3.4-fold induction (0.59% vs. 2.02%); P2: 5.2-fold induction (0.44% vs. 2.32%); P3: 16.2-fold induction (0.14% vs. 2.28%); PaTu8988: 5.1-fold induction (0.54% vs. 2.73%), Fig. 1a). Furthermore, mRNA and protein expression analysis demonstrated that stem-related markers (Nanog, Oct4, Sox2, and C-myc) were markedly increased in CD133^−^ cancer cells following 7 days coculture with irradiated feeder cells (Fig. 1b, c). Consistently, irradiated feeder cells induced CD133^−^ cancer cells to form more spheres, which also presented higher levels of CSC markers CD133 and CD44 (Fig. 1d, e). Together, these results suggest that irradiation-induced cell death promotes CD133^−^ dedifferentiation into CSCs.

### Irradiation induced cell death promotes CD133^−^ cancer cell dedifferentiation via HMGB1-TLR2 interaction

**Fig. 2:**
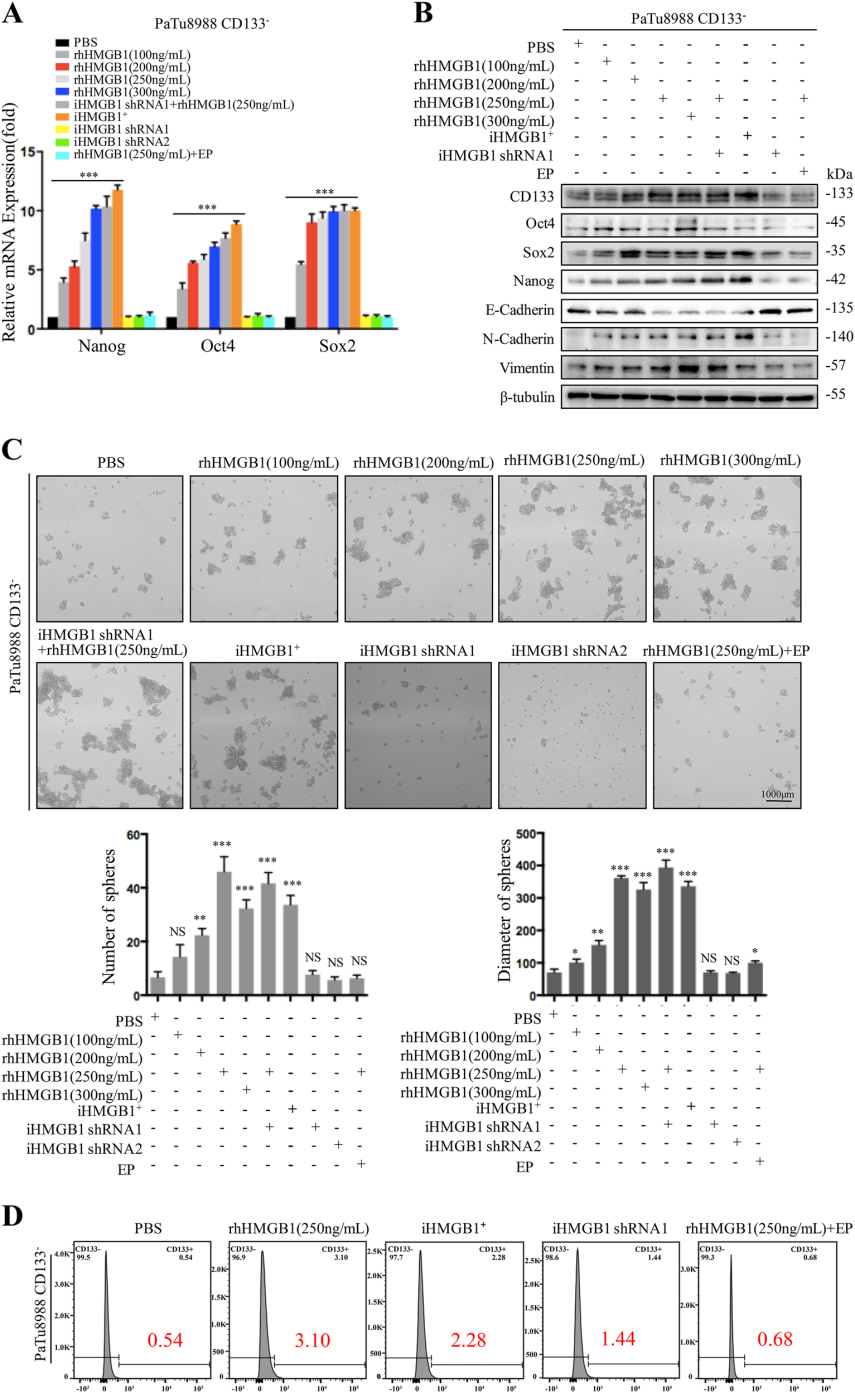
X-ray irradiation induced cell death promotes CD133^−^ dedifferentiation via releasing HMGB1. Sorted CD133^−^ cancer cells (reporter cells) were treated with the following agents: (i) Different concentration of rhHMGB1(100, 200, 250, and 300 ng/mL); (ii) 20 Gy X-ray irradiated HMGB1 wide type cancer cells (iHMGB1^+^); (iii) 20 Gy X-ray irradiated HMGB1 knock down cancer cells (iHMGB1shRNA1 and iHMGB1 shRNA2); (iv) 250 ng/mL rhHMGB1+iHMGB1shRNA1; (v) 250 ng/mL rhHMGB1+EP; (vi) PBS for the indicated time. **a** qRT-PCR analysis the gene expression level of Nanog, Oct4, and Sox2 in CD133^−^ reporter cancer cells following 7 days treatment. **b** Western blot analyses the protein expression level of stem cell and EMT related markers (CD133, Oct4, Sox2, C-myc, Nanog, E-cadherin, N-cadherin, and Vimentin) in CD133^−^ reporter cancer cells following 7d treatment. **c** Formation of sphere number and size were measured in cocultured CD133^−^ reporter cancer cells following 7d treatment. **d** Flow cytometry analysis the percentage of CD133^+^ cells in the CD133^−^ reporter cancer cells following 7 days treatment. Experiments were repeated three times and the data were expressed as mean ± SEM. Student's *t*-test, oneway, two-sided ANOVA. *P < 0.05, **P < 0.01, ***P < 0.001

We previously found that dying cells release HMGB1 to drive an aggressive phenotype in pancreatic cancer^16^. To examine the possible role of dying cell derived HMGB1 in dedifferentiation of resident cancer cells, we silenced HMGB1 using two RNAi in primary and established pancreatic cancer cells, and the silencing efficacy was confirmed by western blot (Fig. S2A). The CD133^−^ cancer cells were then con-cultured with following agents for 7 days: (i) Different concentration of recombinant human HMGB1(rhHMGB1, 100, 200, 250, and 300 ng/mL); (ii) 20 Gy X-ray irradiated HMGB1 wide type cancer cells (iHMGB1^+^); (iii) 20 Gy X-ray irradiated HMGB1 knock down cancer cells (iHMGB1shRNA1); (iv) 20 Gy X-ray irradiated HMGB1 knock down cancer cells (iHMGB1shRNA2); (v) 250 ng/mL rhHMGB1+ iHMGB1shRNA1; (vi) 250 ng/mL rhHMGB1+ethyl pyruvate (EP, HMGB1 inhibitor); and (vii) PBS(control). iHMGB1^+^ resulted in significant upregulation of mRNA and protein expression of stem cell markers (Oct4, Sox2, and Nanog) in CD133^−^ cancer cells, their sphere-forming ability, as well as CD133^+^ cell percentage 7 days post coculture, which were comparable to 250–300 ng/mL rhHMGB1 (Fig. 2a–d). In contrast, inhibiting HMGB1 expression (iHMGB1shRNA1 and iHMGB1shRNA2) in feeder cells or activity (EP) attenuated these effects (Fig. 2a–d and Fig. S3A), supporting our hypothesis that HMGB1 released from irradiated cancer cells positively mediates this dedifferentiation process. It is well known that EMT process can induce cancer cell dedifferentiation into CSCs. We also further confirmed that CD133^−^ cancer cells exposure to the HMGB1^+^ irradiated cells or rhHMGB1 led to acquisition of higher migration ability (Fig. S2B) and mesenchymal phenotype accompanied by up-regulation of mesenchymal makers (N-cadherin and Vimentin) and down-regulation of epithelial marker (E-cadherin) (Fig. 2b and Fig. S3A).

**Fig. 3:**
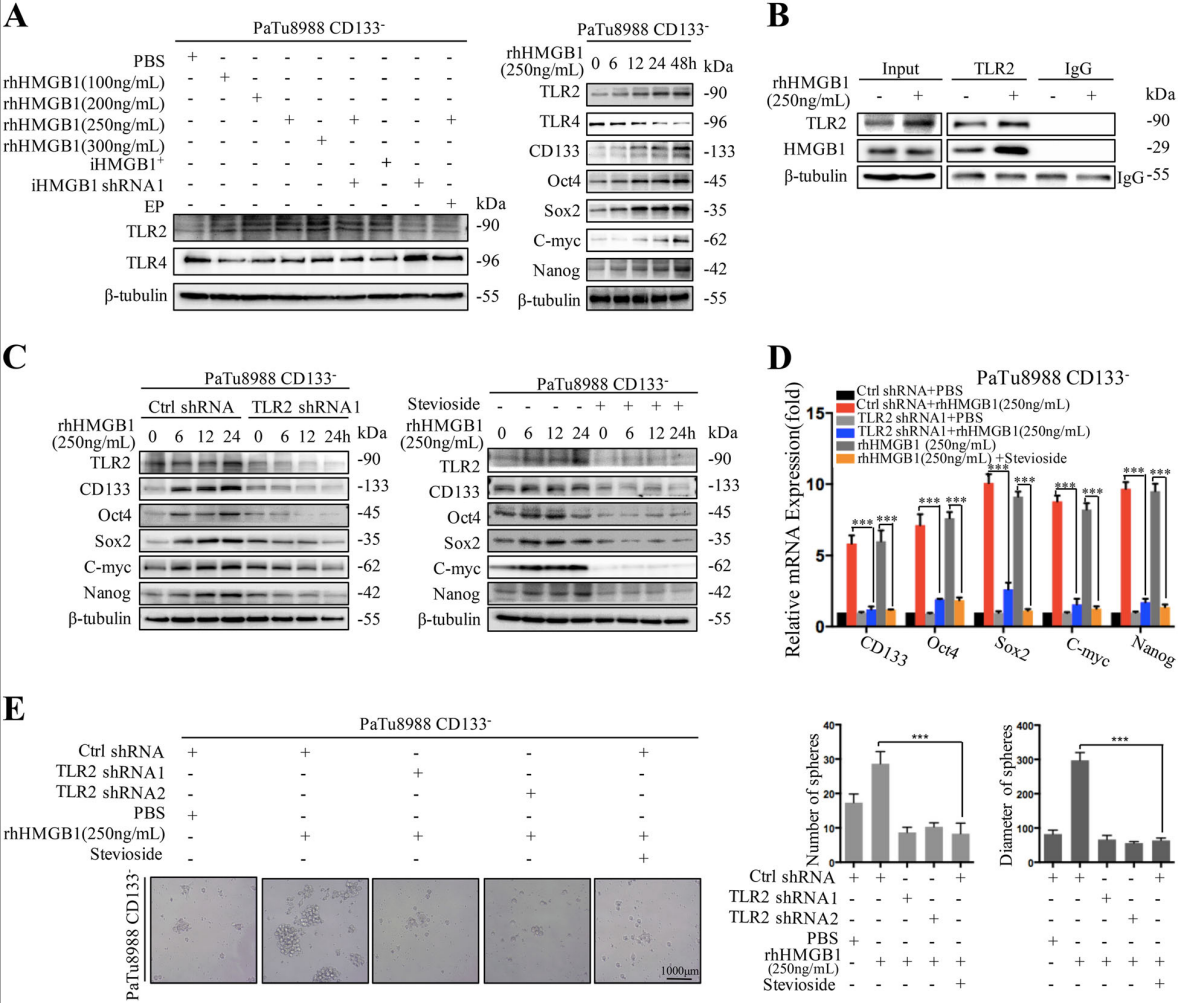
HMGB1 maintains and enhances the stemness of CD133^−^ cancer cells depending on TLR2. Sorted CD133^−^ cancer cells (reporter) were treated with the following agents: (i) Different concentration of rhHMGB1 (100, 200, 250, and 300 ng/mL); (ii) 20Gy X-ray irradiated HMGB1 wide type cancer cells (iHMGB1^+^); (iii) 20Gy X-ray irradiated HMGB1 knock down cancer cells (iHMGB1 shRNA1); (iv) 250 ng/mL rhHMGB1+iHMGB1shRNA1; (v) 250 ng/mL rhHMGB1+EP; (vi) PBS for the indicated time. **a** Western blot analyses the expression of TLR2 and TLR4 in treated CD133^−^ cancer cells for the indicated time (0, 6, 12, 24, and 48 h). **b** Co-IP analyses the binding between HMGB1 and TLR2 in CD133^−^ cancer cells. **c**, **d** Sorted CD133^−^ cancer cells treated with rhHMGB1 (250 ng/ml) and with or without Stevioside (TLR2 inhibitor) or sh-TLR2 silenced CD133^−^ cancer cells for the indicated time (0, 6, 12, and 24 h). qRT-PCR and Western blot analyses the expression of stem cell-related markers (CD133, Oct4, Sox2, C-myc, and Nanog) at protein and gene level. **e** Sphere forming ability of sorted CD133^−^ cancer cells or sh-TLR2 silenced CD133^−^ cancer cells treated with rhHMGB1 (250 ng/ml) and with or without Stevioside. Experiments were repeated three times and the data were expressed as mean ± SEM. Student's *t*-test, oneway, and two-sided ANOVA. *P < 0.05, **P < 0.01, ***P < 0.001

Assessing putative cell surface receptors (TLR2 and TLR4) binding with extracellular HMGB1, we found that TLR2, but not TLR4, was upregulated in the CD133^−^ cancer cells when treated with HMGB1^+^ irradiation cells or rhHMGB1 (Fig. 3a). TLR2 and stem cell-related markers (CD133, Oct4, Sox2 C-myc, and Nanog) were evaluated in a time and concentration dependent manner with rhHMGB1 (Fig. 3a). Co-IP assay further confirmed the interaction between HMGB1 and TLR2 when CD133^−^ cancer cells were cocultured with the 250 ng/ml rhHMGB1 (Fig. 3b). To study the functional consequences of TLR2 in HMGB1 mediating CD133^−^ cancer cells dedifferentiation, TLR2 was inhibited by shRNA technology or used the TLR2 inhibitor (Stevioside) in the CD133^−^ cancer cell coculture system. TLR2 inhibition exhibited pronounced suppression of stem cell markers induced by HMGB1 (Fig. 3c, d, and Fig. S3B). Consistently, HMGB1 promoted CD133^−^ cancer cells sphere forming ability was also abrogated following suppress the expression and function of TLR2 (Fig. 3e). In summary, these results indicate that extracellular HMGB1 interacts with TLR2 receptor on CD133^−^ cancer cells to promote their dedifferentiation into CSCs.

### HMGB1-TLR2 induced CD133^−^ cancer cells dedifferentiation via regulating Hippo-YAP pathway

**Fig. 4:**
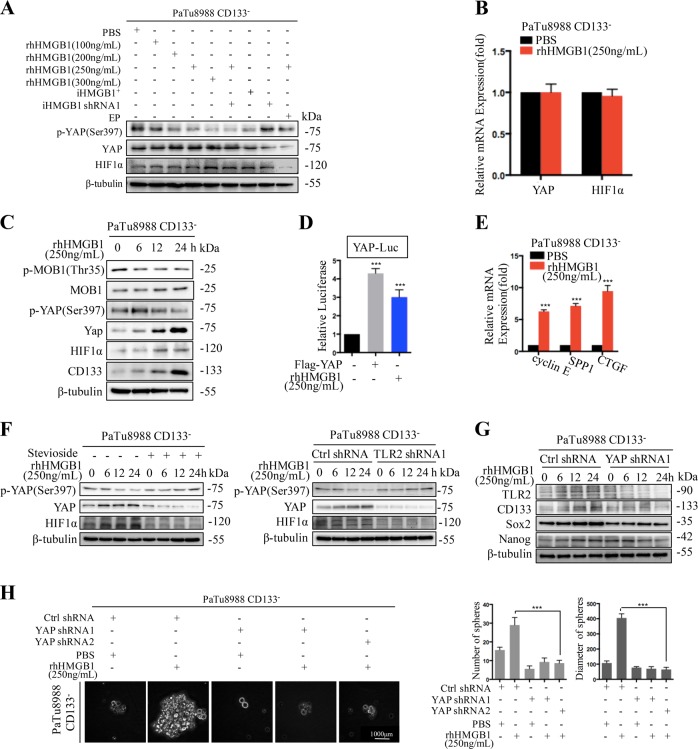
HMGB1-TLR2 induced CD133^−^ cancer cells dedifferentiation via regulating Hippo-YAP pathway. **a** Western blot analyses the expression of YAP, p-YAP and HIF-1α in sorted CD133^−^ cancer cells (reporter cells) treated with the following agents: (i) Different concentration of rhHMGB1 (100, 200, 250, and 300 ng/mL); (ii) 20Gy X-ray irradiated HMGB1 wide type cancer cells (iHMGB1^+^); (iii) 20 Gy X-ray irradiated HMGB1 knock down cancer cells (iHMGB1 shRNA1); (iv) 250 ng/mL rhHMGB1+iHMGB1shRNA1; (v) 250 ng/mL rhHMGB1+ EP; (vi) PBS for the indicated time. **b** qRT-PCR analyses the expression of YAP and HIF-1α in cocultured CD133^−^ reporter cell population. **c** Western blot analyses the expression of Hippo-YAP pathway related marker (p-MOB1, MOB1, p-YAP, and YAP), HIF-1α, and CD133 in CD133^−^ cancer cells treated with rhHMGB1 (250 ng/mL) for the indicated time (0, 6, 12, and 24 h). **d** Relative YAP luciferase activity in sorted CD133^−^ cancer cells in the presence and absence of rhHMGB1 or Flag-YAP. **e** qRT-PCR analyses the expression of YAP downstream target genes (cyclin E, SPP1, and CTGF) in CD133^−^ cell population in the presence or absence of rhHMGB1. **f** Western blot analyses the expression of p-YAP, YAP, and HIF-1α in CD133^−^ cancer cells or sh-TLR2 silenced CD133^−^ cancer cells treated with rhHMGB1(250 ng/ml) and with or without Stevioside. **g** Western blot analyses the expression of TLR2, CD133, Sox2, and Nanog in CD133^−^ cancer cells and YAP silenced CD133^−^ cancer cells treated with rhHMGB1 (250 ng/ml). **h** Sphere forming ability of sorted CD133^−^ cancer cells or YAP silenced CD133^−^ cancer cells treated with or without rhHMGB1(250 ng/ml). Experiments were repeated three times and the data were expressed as mean ± SEM. Student's *t*-test, oneway, and two-sided ANOVA. *P < 0.05, **P < 0.01, ***P < 0.001

To identify downstream effectors of HMGB1-TLR2 mediated cancer cell dedifferentiation, we examined the expression levels of several key signaling pathways regulating cancer cell plasticity, and noticed the protein expression of YAP and MOB1 (core component of the Hippo pathway) was slightly enhanced upon rhHMGB1 stimulation (Fig. 4a). Interestingly, we found YAP phosphorylation(Ser397), which is associated with YAP protein degradation, was significantly reduced in CD133^−^ cancer cells treated with HMGB1^+^ irradiated cells or rhHMGB1 (Fig. 4a, c). YAP mRNA expression was not affected regardless of HMGB1 presence, further suggesting HMGB1 enhanced YAP protein expression by inhibiting YAP phosphorylation (Fig. 4b). HMGB1decreased the YAP phosphorylation in a time dependent manner at the peak inhibition on 6 h post treatment, in accompany with enhanced expression of YAP (Fig. 4c). Luciferase assay revealed that YAP luciferase activity was promoted (Fig. 4d). Consistently, mRNA expression levels of YAP target genes such as cyclin E, SPP1, and CTGF were up-regulated in CD133^−^ cancer cells following rhHMGB1 treatment (Fig. 4e). YAP silencing reduced the expression of stem cell-related markers (CD133, Nanog, Sox2), as well as sphere-forming ability of rhHMGB1 stimulated CD133^−^ cancer cells (Fig. 4g, h, Fig. S3C), confirming its role in cancer cell stemness. Furthermore, TLR2 silencing enhanced the activation of YAP(the rational of ratio of phosphorylation YAP to total YAP increased) in HMGB1 stimulated CD133^−^ cancer cells (Fig. 4f, Figs. S3B and S4), suggesting that YAP is downstream of HMGB1-TLR2 signaling to induce CD133^−^ cancer cells dedifferentiation.

### YAP/HIF-1α complex contribute to HMGB1 induced CD133^−^ cancer cells dedifferentiation

In addition to acting as an oncogene in many cases, YAP may complex with other transcription factors to regulate cancer biology including HIF-1α, which controls cancer cells stemness following radiotherapy in an oxygen dependent or independent manner. We found that protein instead mRNA level of HIF-1α rose in CD133^−^ cancer cells following HMGB1 treatment in normoxia (Fig. 4a–c). Knockdown of TLR2 or YAP in CD133^−^ cancer cells decreased HIF-1α expression under normoxia condition (Fig. 4f), suggesting HIF-1α is downstream of TLR2 and YAP.

**Fig. 5:**
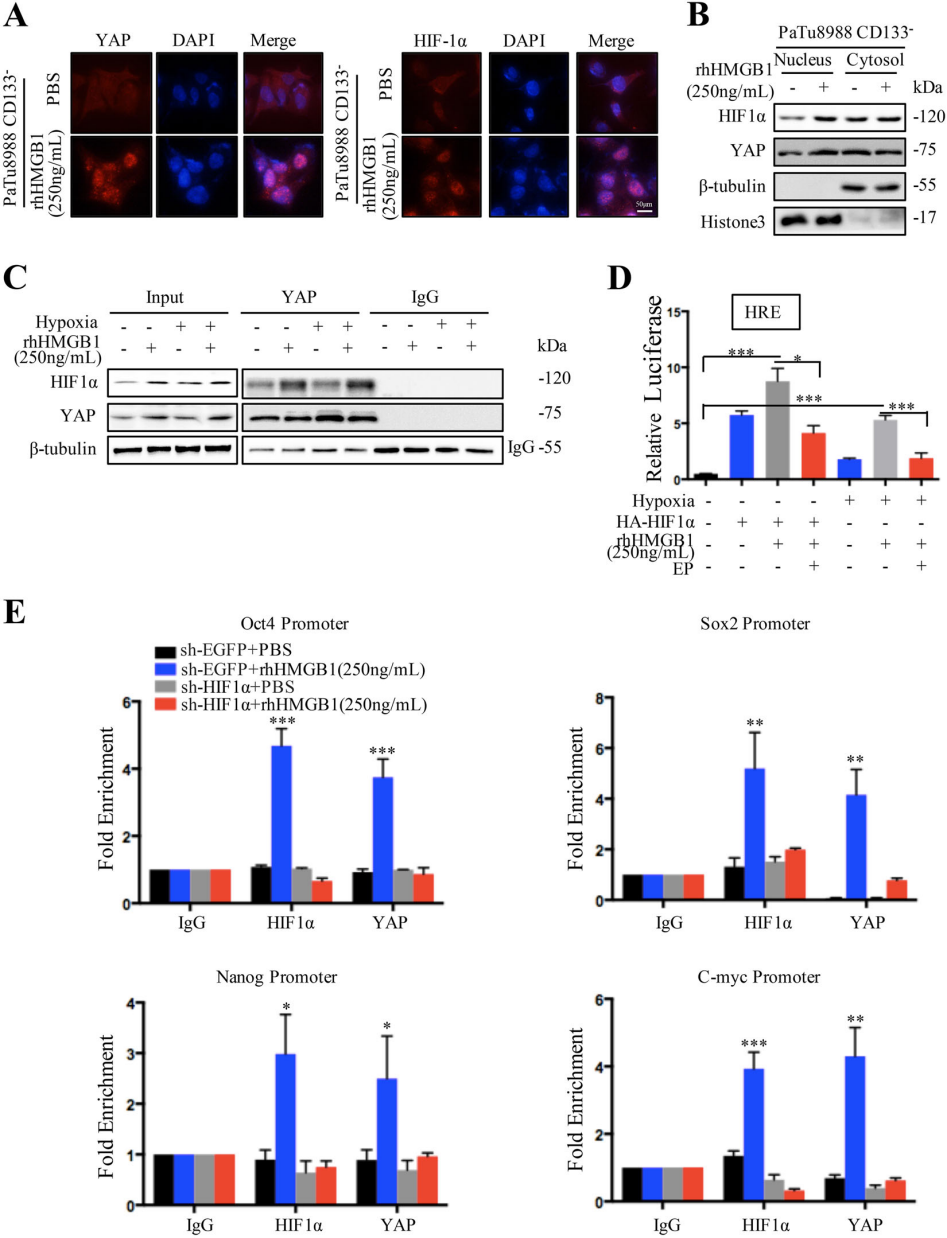
YAP/HIF-1α complex contribute to HMGB1 induced CD133^−^ cancer cells dedifferentiation. **a** Immunofluorescence staining accessed the localization of YAP and HIF-1α in CD133^−^ cancer cells treated with or without rhHMGB1. **b** Western blot analyses the expression of YAP and HIF-1α in the cytoplasm and nuclear of CD133^−^ cancer cells treated with or without rhHMGB1. **c** Co-IP analysis of the YAP/HIF-1α complex forming in the CD133^−^ cancer cell population treated with or without rhHMGB1 under hypoxia or normoxia condition. **d** Relative HRE luciferase activity in CD133^−^ cancer cells treated with or without HA-HIF-1α/rhHMGB1/EP under hypoxia or normoxia condition. **e** ChIP assay accessed HIF-1α band to site of pluripotent genes promoter genomic DNA sequence treated with or without rhHMGB1 in CD133^−^ cancer cells and HIF-1α knockdown CD133^−^ cancer cells. Experiments were repeated three times and the data were expressed as mean ± SEM. Student's *t*-test, oneway, and two-sided ANOVA. *P < 0.05, **P < 0.01, ***P < 0.001

To further confirm the relationship between YAP and HIF-1α, we first examined the localization of YAP and HIF-1α in CD133^−^ cancer cells following rhHMGB1 treatment by immunofluorescence. YAP and HIF-1α can be detected in CD133^−^ cancer cells with or without rhHMGB1 treatment. YAP and HIF-1α were primarily detected in the cytoplasm of cancer cells without HMGB1 treatment, while displayed distinct nuclear localization following rhHMGB1 treatment (Fig. 5a). Western blot also confirmed that YAP and HIF-1α nuclear translocation enhanced following rhHMGB1 treatment (Fig. 5b). Moreover, YAP nuclear translocation was significantly inhibited in HIF-1α knockdown CD133^−^ cancer cells in the presence of rhHMGB1 treatment (Fig. S3E). YAP-HIF-1α complex was observed in CD133^−^ cancer cells treated with rhHMGB1 in both normoxia and hypoxia (Fig. 5c) by co-IP assay. YAP luciferase activity in presence of HIF-1α was promoted in rhHMGB1 treated CD133^−^ cancer cells. Moreover, HRE luciferase activity, indicated HIF-1α DNA binding ability, was enhanced in HMGB1 treated CD133^−^ cancer cells and suppressed in the presence of EP under hypoxia (Fig. 5d).

To investigate whether HIF-1α directly regulates pluripotent genes (Oct4, Sox2, C-myc, and Nanog) expression, we searched the promoter regions of these genes and identified site consensus to HIF-1α binding sequences (Fig. S5A). ChIP assay revealed that HMGB1 promoted HIF-1α binding to promoter regions of these pluripotent genes, while the binding ability was abolished in HIF-1α knockdown cells in presence of rhHMGB1 (Fig. 5e). To further confirm that HIF-1α is required for the recruitment of YAP, we assessed if HMGB1 promoted YAP binding to pluripotent genes promoter regions (Fig. S5B) by CHIP in HIF-1α knockdown and control CD133^−^ cancer cells. The results revealed that HMGB1 promoted YAP binding to pluripotent gene promoter regions in the presence of HIF-1α, while the binding ability was abolished in HIF-1α knockdown cells in presence of rhHMGB1 (Fig. 5e, Fig. S5B). Taken together, these data indicate that dying cells derived HMGB1 mediated formation of nuclear YAP-HIF1α complex, which further activates expression of the pluripotency factors.

### HMGB1-TLR2-YAP/HIF-1α induced CD133^−^ cancer cell dedifferentiation in vivo

**Fig. 6:**
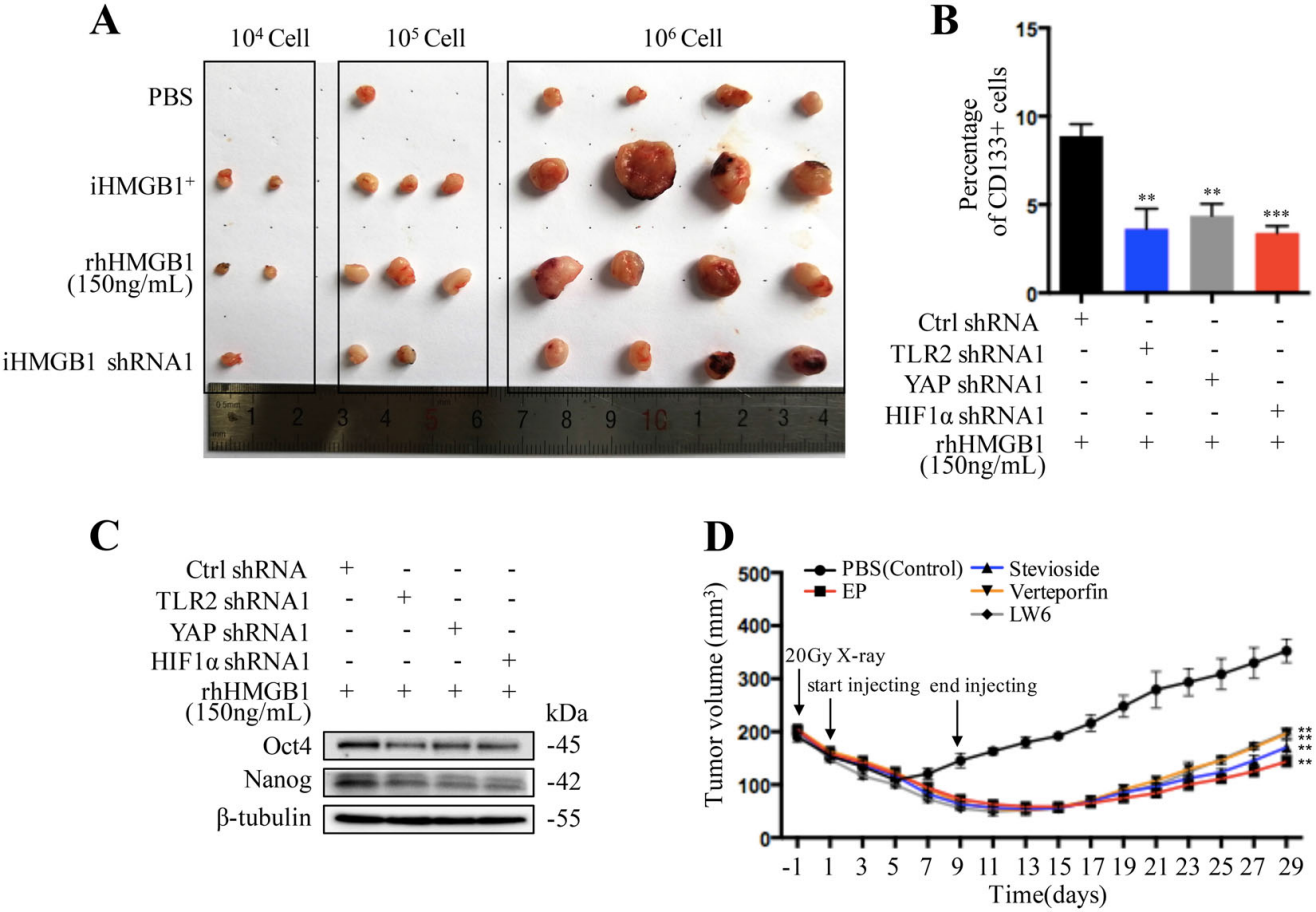
HMGB1-TLR2-YAP/HIF-1α induced CD133^−^ cancer cells dedifferentiation in vivo. **a** CD133^−^ cancer cells were cocultured with irradiated parental cancer cells HMGB1^+^ cells (HMGB1 positive, HP), irradiated HMGB1 knock down cancer cells (iHMGB1shRNA1, HN), rhHMGB1 (150 ng/mL), and the same empty medium (PBS). The tumor initiating capacity and volume were measured. **b**, **c** CD133^−^ PaTu8988 cancer cells, TLR2, YAP, or HIF-1α knockdown PaTu8988 CD133^−^ cancer cells were implanted subcutaneously into the right dorsal flanks of nude mice, respectively and treated with rhHMGB1 for 2 weeks. Flow cytometry analysis the percentage of CD133^+^ cancer cells (**b**). Western blot analyses the protein expression level of Nanog and Oct4 in the fresh tumor tissues (**c**). **d** The subcutaneous tumor mice models were received 20Gy X-ray irradiation and injected of PBS, EP (HMGB1 inhibitor), Stevioside (TLR2 inhibitor), Verteporfin (YAP inhibitor), or LW6 (HIF-1α inhibitor). The tumor volume and growth speed were measured. Experiments were repeated three times and the data were expressed as mean ± SEM. Student's *t*-test, oneway, two-sided ANOVA. *P < 0.05, **P < 0.01, ***P < 0.001

In order to confirm the molecular mechanisms of HMGB1 inducing cancer cell dedifferentiation in vivo, we firstly determined the tumor initiation potential of CD133^−^ cancer cells in similar in vitro conditions. Sorted CD133^−^ PaTu8988 cancer cells were cocultured with irradiated parental cancer cells (iHMGB1^+^ cells), irradiated HMGB1 knockdown cancer cells (HMGB1^−^ cells), rhHMGB1 (150 ng/mL), or normal medium (control group) and then injected subcutaneously into nude mice for the indicated cell number (1 × 10^4^, 1 × 10^5^ and 1 × 10^6^ cells/mouse were injected, respectively). CD133^−^ cancer cells cocultured with iHMGB1^+^ cells and rhHMGB1 showed significantly higher tumor initiating capacity than the other groups. 4/4 mice developed tumors in each group until the cell number reached 1 × 10^6^, but tumors derived from HMGB1^+^ cells and rhHMGB1 coculture group were larger than the other groups (Fig. 6a).

To confirm the molecular mechanisms in vivo, 1 × 10^6^ CD133^−^ PaTu8988 cancer cells, TLR2, YAP, or HIF-1α knockdown PaTu8988CD133^−^ cancer cells were implanted subcutaneously into the right dorsal flanks of nude mice, respectively. When the tumors reached a volume of 200 mm^3^, the mice were treated with rhHMGB1 by peritumoral injection every 2 days for 2 weeks, and tumors were harvested 3 days after the last injection for analysis of stemness. HMGB1 treatment resulted in significant upregulation of stem cell markers (Oct4 and Nanog) and CD133^+^ cell proportion in fresh tumor tissues, which were abrogated by silencing TLR2, YAP, or HIF-1α (Fig. 6b, c). As a comparison to rhHMGB1 treatment, the mice received peritumoral injections of PBS, EP (HMGB1 inhibitor), Stevioside (TLR2 inhibitor), Verteporfin (YAP inhibitor), or LW6 (HIF-1α inhibitor) every 2 day for 2 weeks. Combination treatment of X-ray irradiation with TLR2-YAP-HIF-1α pathway inhibitors prevented tumor relapse (Fig. 6d), indicating that inhibiting the HMGB1-TLR2-YAP-HIF-1α pathway blocked radiotherapy-induced dedifferentiation of primary tumor cells and also prevents tumor relapse.

### Radiation-induced cancer cell death constitute a supporting niche for cancer cell stemness

**Fig. 7:**
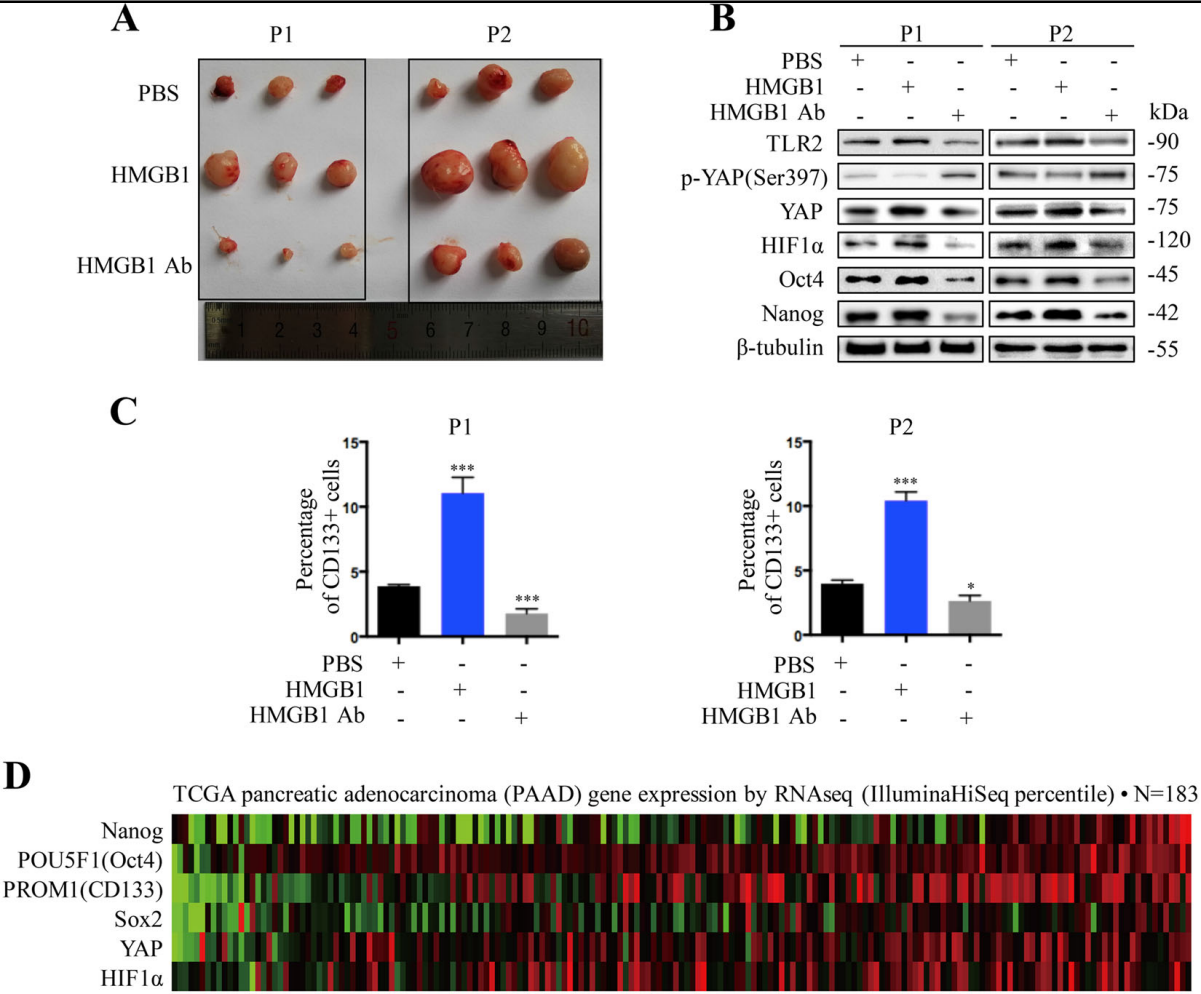
Radiation-induced cancer cell death constitute a supporting niche for cancer cell stemness. **a** Established PDXs (HPCx1 and HPCx2) model received 20 Gy X-ray irradiation and then treated with PBS, rhHMGB1 and HMGB1 antibody. The tumor volume was measured. **b** Western blot analyses the expression of TLR2, p-YAP, YAP, HIF-1α, Nanog, and Oct4 in the fresh tumor tissues. **c** Flow cytometry analysis the percentage of CD133^+^ cancer cells from the fresh tumor tissues. **d** Analysis of the TCGA database indicates that the gene expression level of Nanog, CD133, Oct4, Sox2, YAP, and HIF-1α in pancreatic adenocarcinoma. The results are presented by heat map (*n* = 183). Statistical analysis was determined by Pearson correlation analysis

Using two established patient derived xenograft (PDX) models (HPCx1 and HPCx2), we explored the role of HMGB1 in mediating the tumor relapse. The mice received 20 Gy X-ray irradiation, and were randomized 7 days later to receive surrounding tumor injections of PBS, HMGB1, and HMGB1 antibody every 2 day for 2 weeks. The experiment was terminated on 50 days upon treatment. HMGB1 treatment promoted tumor regrowth (Fig. 7a) and increased TLR2-YAP-HIF-1α signaling activation compared with the parental control (Fig. 7b). Moreover, the HMGB1 treated model has a higher percentage of CD133^+^ cancer cells, which were abrogated by HMGB1 antibody treatment (Fig. 7c). Using the information from TCGA database (https://genome-cancer.ucsc.edu, *n* = 183), we evaluated the correlation gene expression of CD133, Oct4, Nanog, Sox2, YAP, and HIF-1α, and the heat-map revealed a linear relationship between the expression of these markers in TCGA protein array (Fig. 7d).

## Discussion

**Fig. 8:**
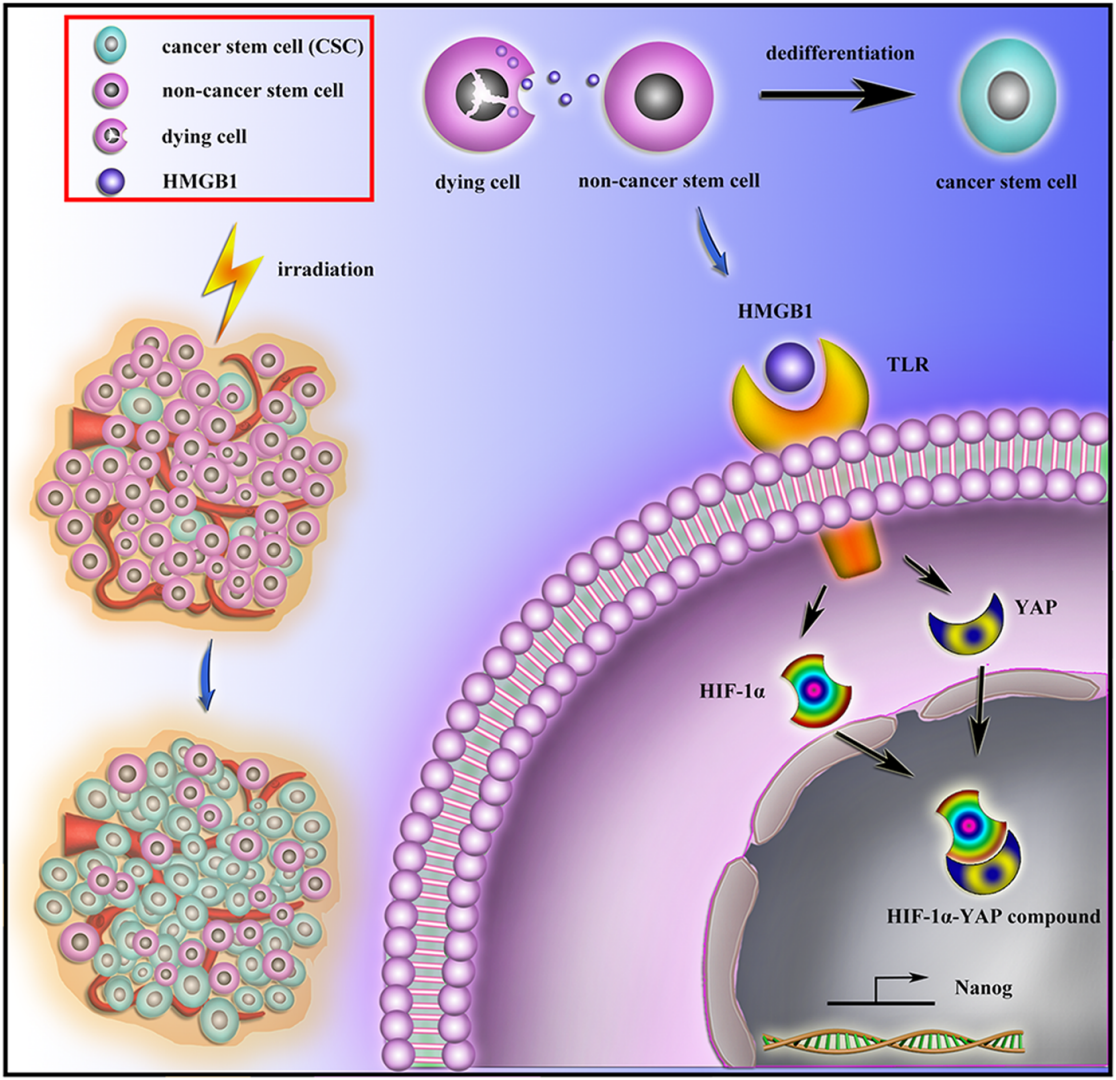
Schematic of process driven by radiotherapy-induced HMGB1/TLR2/YAP/ HIF-1α signaling driven pancreatic cancer dedifferentiation and stemness. Radiotherapy induced the cancer cells’ death that enriched the cancer stem cells. Dying cells released HMGB1 binds with TLR2 expressed on resident pancreatic cancer cells. HMGB1/TLR2 stimulates YAP/HIF-1α in a paracrine manner, which driven the pancreatic cancer cells dedifferentiation and stemness process

In this study, we confirmed CD133^−^ pancreatic cancer cells dedifferentiation to CSCs using primary human pancreatic carcinoma tissues, established pancreatic cancer cell line, and a PDX model following radiotherapy. Furthermore, dying cell derived HMGB1 activates TLR2/YAP/HIF-1α pathways and induces neighboring CD133^−^ pancreatic cancer cells dedifferentiation, which is an important signaling event underlying tumor relapse following radiotherapy (Fig. 8). Therefore, blocking this pathway may prevent both enrichment of CD133^+^ cancer stem cell population and tumor recurrence.

The resulting cell death may also stimulate the generation of DAMPs and their corresponding pattern recognition receptors. As one most common DAMPs, HMGB1 is complicated in tumor treatment resistant by its paradoxical dual activities. Huang et al. reported that HMGB1 participated in immune-scavenging in advanced rectal cancer that have undergone neoadjuvant chemo- or radiotherapy^20^. Yin et al. found that HMGB1-mediated autophagy attenuates gemcitabine-induced apoptosis in bladder cancer cells^21^. Extracellular HMGB1 on cancer cell stemness has gathered increasing attention. Cancer-associated fibroblasts derived HMGB1 promoted stemness and tumourigenicity in breast cancer^22^. HMGB1 is also found to act as a potent EMT driver in colorectal carcinoma and gastric cancer^23,24^. It has confirmed that cancer cells undergoing EMT acquire stem cell like properties^25^. These researches indirectly confirmed the role of HMGB1 in cancer cells plasticity. Our results for the first time supply the direct evidence that dying cells derived HMGB1 induced the CD133^−^ cancer cells dedifferentiation in pancreatic cancer following radiotherapy. This multi-activity of extracellular HMGB1 in the regulation of the cancer cell biology may depend on its secretory manner, redox status, cleavage, and special binding receptors. Our results also confirmed that both HMGB1 redox state and oxidized state promotes CD133^−^cancer cells dedifferentiation (Fig. S6). Knockdown HMGB1 in the feeder cancer cells can not completely abrogate this effect. This can be ascribed to: (i) some other inflammatory factors (such as IL-1β and TNF-a) released from the irradiation cell to the culture media have the similar function; (ii) FACS-purified cell population is not 100% pure. Indeed, we found sorted CD133^−^ cancer cells always contained a very small population of contaminating CD133^+^ cancer cells.

YAP is overexpressed in many cancers and plays important roles in cancer cell proliferation, metabolism, and treatment resistance^26^. Ectopic expression of YAP has been recently associated with cancer cell plasticity. Panciera et al. found that transient expression of YAP could converts differentiated cells (neurons and pancreatic exocrine cells) into cells displaying multiple features of their corresponding tissue-specific stem cells^27^. In breast cancer, YAP/TAZ activity is tightly linked with reprogram non-cancer stem cells into CSCs^28^. In hepatocellular carcinoma, elevated YAP activity was a key step conferring cancer cell with stem-like properties, including chemoresistance and tumorigenicity, under continuous 5-FU treatment^29^. In this study, we found that dying cells-released HMGB1 promoted pancreatic cancer cells dedifferentiation by decreasing YAP phosphorylation and triggering YAP nuclear translocation. Furthermore, YAP knockdown significantly decreased HIF-1α expression and inhibited HMGB1/TLR2 induced CD133^−^ cancer cell dedifferentiation. YAP mainly dependent on multiple domains to interact with transcription factors and form a complex to promote the expression and activation of downstream target genes in the nucleus. HIF-1α could be activated in a hypoxia dependent and independent manner following radiotherapy^30^. Recent studies have demonstrated that HIF-1α was required for the maintenance of CSCs in response to hypoxia or chemotherapy^31,32^. Dai et al. also reported that HIF-1α did not mediate hypoxia-triggered YAP nuclear translocation in hepatocellular carcinoma cells^33^. Zhang et al. shown that YAP interacts directly with HIF-1α in the nucleus and sustains HIF-1α stability, which further correlated positively with hepatocellular carcinoma progression. Our data are in line with the later observations to show that HMGB1 triggered both YAP and HIF-1α nuclear translocation and enhanced YAP and HIF-1α interaction. As silencing YAP decreased HIF-1α protein expression, it is possible that YAP is important for HIF-1α stability in hypoxia independent manner under HMGB1/TLR2 treatment. Moreover, silencing HIF-1α decreased the YAP nuclear translocation. Thus, HIF-1α is required for YAP performing the transcription factor functional under HMGB1/TLR2 treatment. Recently several studies reported that Nanog was sufficient to confer cancer cells certain CSC properties and phenotypes in prostate and brain cancer^34,35^. From our in vitro study, it was obviously found that Nanog is the most obviously changed gene in the process of CD133^−^ pancreatic cancer cells dedifferentiation compare to the other target gene.

In this study, we found that HMGB1-TLR2 mediated cytoplasmic YAP translocation into the nucleus. Consequently, YAP and HIF-1α form complex in the nucleus and further induce CD133^−^ cancer cells dedifferentiation. Based on our results, direct inducing cancer cell death or targeting of CSCs may not be sufficient to cure cancer. Tumor microenvironmental, such as the paracrine signaling loops between dying cancer cells and resident differentiated cancer cells, could represent the basis of new, innovative treatments target. Overall, we propose a novel strategy combining HMGB1 inhibitors with irradiation for synergistic pancreatic carcinoma treatment.

## Supplementary information

Supplementary Information
