# Supplementary material for: Dedifferentiation process driven by radiotherapy-induced HMGB1/TLR2/YAP/HIF-1α signaling enhances pancreatic cancer stemness
Source: Cell Death Dis. 2019 Sep 26;10(10):724. doi: 10.1038/s41419-019-1956-8 (PMC6763460; doi:10.1038/s41419-019-1956-8)
Supplement: Supplementary file 1 — Supplementary Information [file 41419_2019_1956_MOESM1_ESM.docx]

Supplementary Information for:

Dedifferentiation process driven by radiotherapy-induced HMGB1/TLR2/YAP/ HIF-1α signaling enhances pancreatic cancer stemness

Lirong Zhang^1#^, Hui Shi^1#^, Hongbo Chen^2#^, Aihua Gong^3^, Yanfang Liu^4^, Lian Song^1^, Xuewen Xu^1^, Tao You^1^, Xin Fan^1^, Dongqing Wang^1*^, Fang Cheng^1,2,5*^, Haitao Zhu^1*^


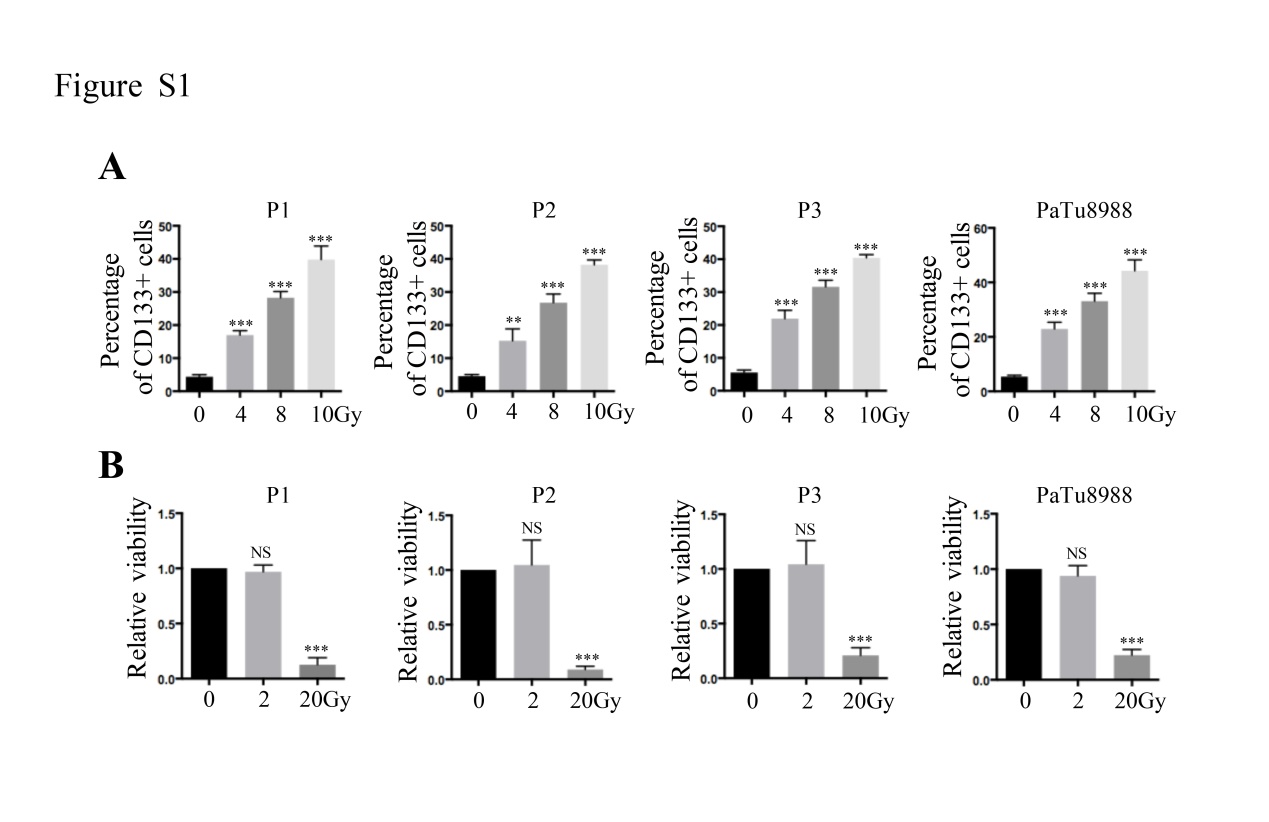


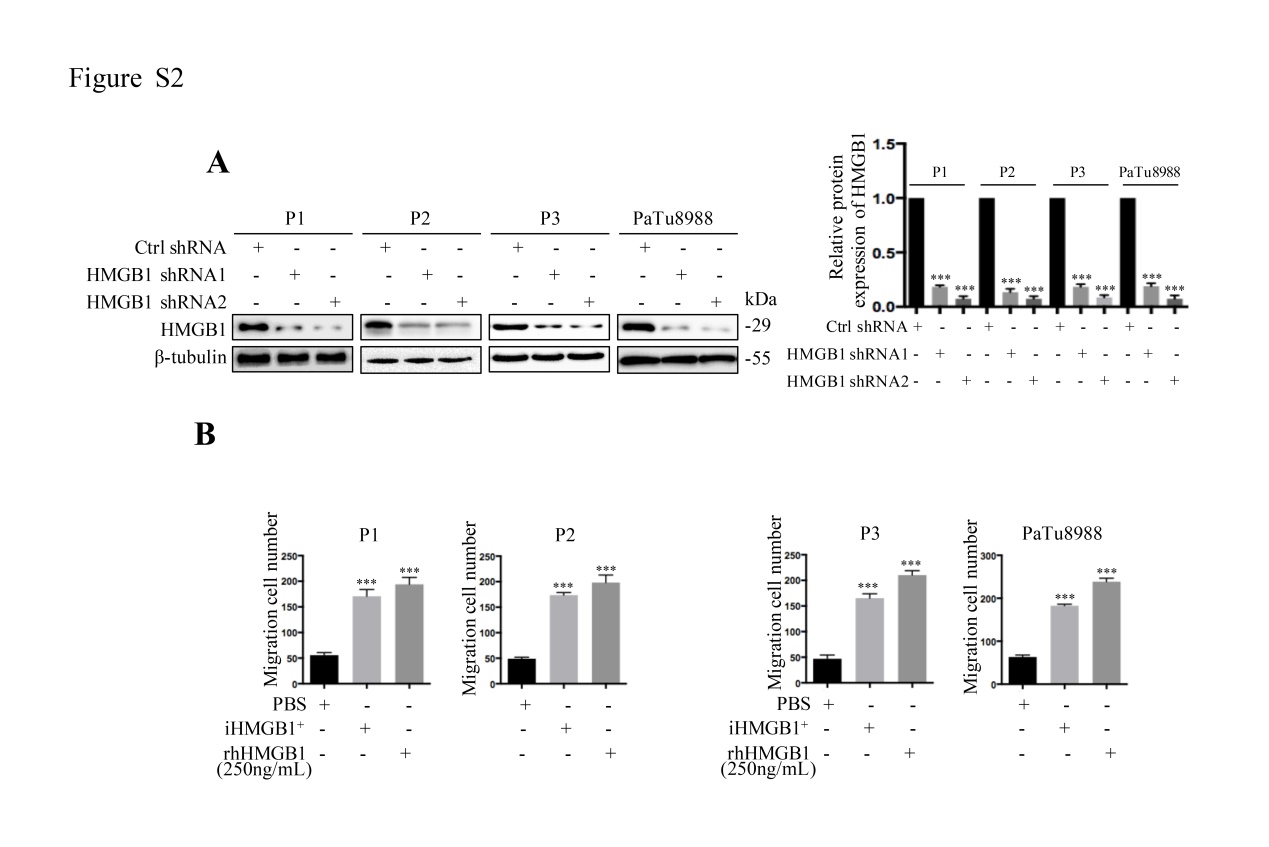


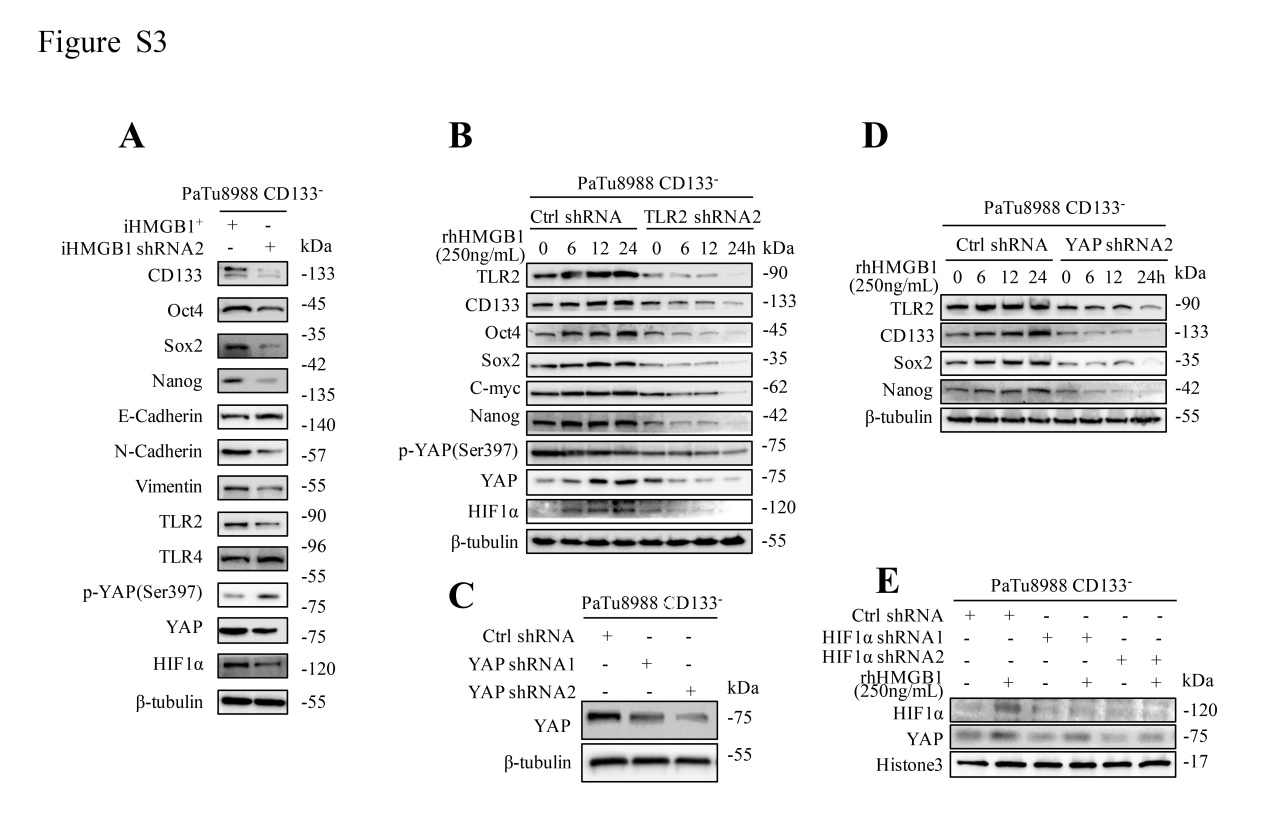


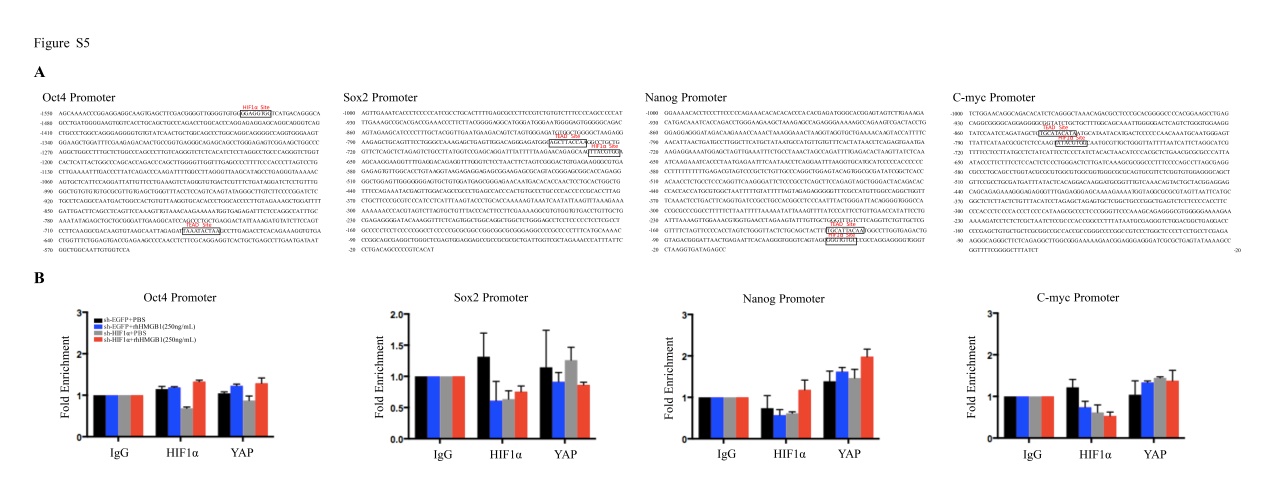


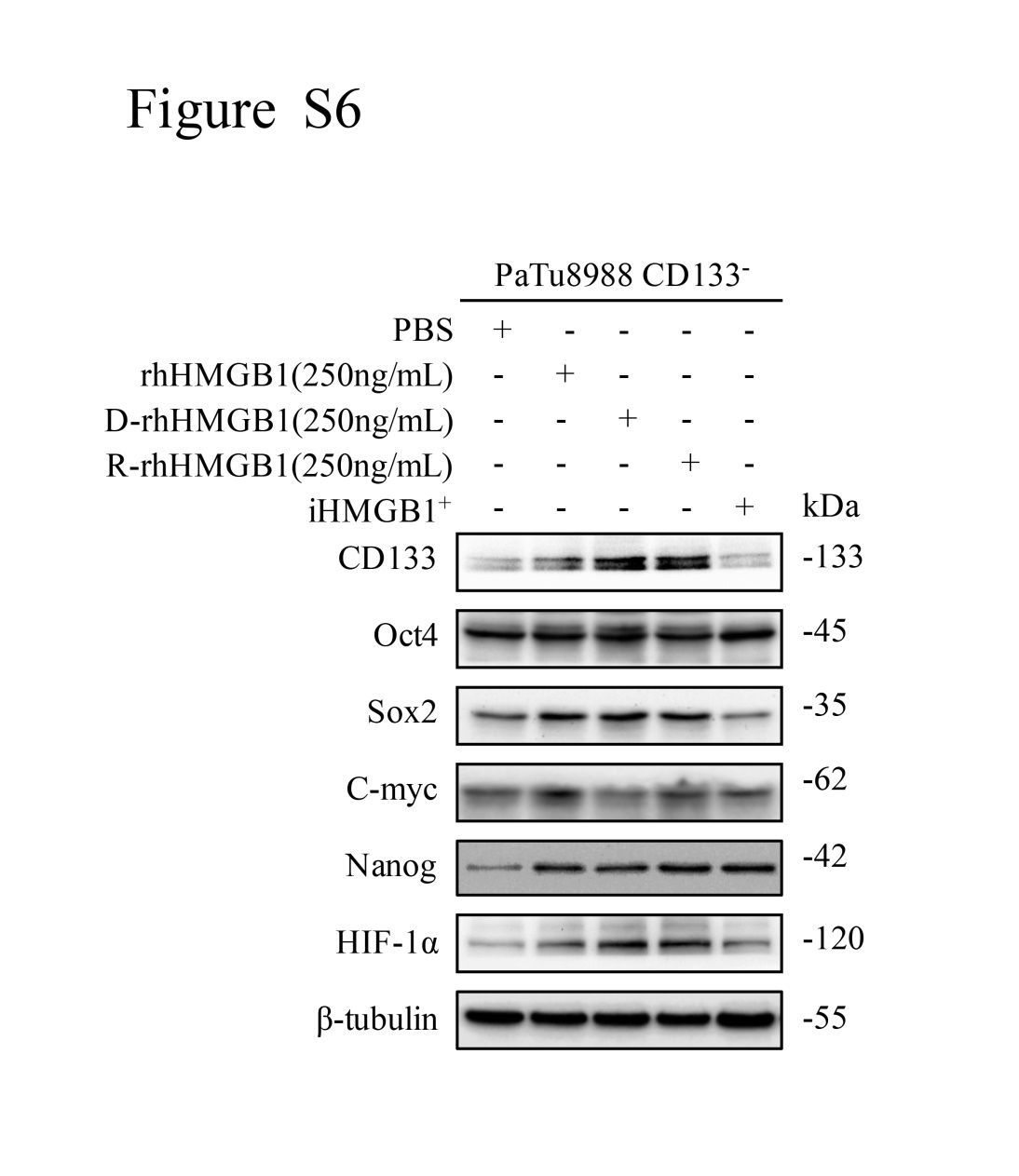


**Figure legend**

**Figure S1** (A) Primary pancreatic cancer cells from pancreatic carcinoma patients (P1, P2 and P3) and established pancreatic cancer cell line (PaTu8988) were irradiated with clinically relevant doses (0, 4, 8, 10Gy) *in vitro*. Flow cytometry analysis the percentage of CD133^+^ cancer stem-like cells in the total population post 7 days irradiation. (B) Effective of 2Gy and 20Gy X-ray irradiation on cancer cells death in vitro. CCK8 accessed cancer cells viability following 3d 2Gy and 20Gy X-ray irradiation. Experiments were repeated three times and the data were expressed as mean±SEM. Student's t-test, Oneway, two-sided ANOVA. **P*<0.05, ***P*<0.01, ****P*<0.001.

**Figure S2** (A) Western blot analyses of shRNA knockdown efficiency of HMGB1 protein in pancreatic cancer cells. (B)Primary pancreatic cancer cells from pancreatic carcinoma patients (P1, P2 and P3) and established pancreatic cancer cell line (PaTu8988) were treated with PBS, iHMGB1 and rhHMGB1 (250ng/ml) *in vitro* for 48h. Transwell assay analysis the migration ability of cancer cells. Experiments were repeated three times and the data were expressed as mean±SEM. Student's t-test, Oneway, two-sided ANOVA. **P*<0.05, ***P*<0.01, ****P*<0.001.

**Figure S3** (A) Western blot analyses the expression of CD133, Oct4, Sox2, Nanog, E-Cadherin, N- Cadherin, Vimentin, TLR2, TLR4, p-YAP(Ser397), YAP and HIF-1α in CD133^-^PaTu8988 cancer cells treated : i) 20Gy X-ray irradiated HMGB1 wide type cancer cells (iHMGB1^+^); iii) 20Gy X-ray irradiated HMGB1 knock down cancer cells (iHMGB1 shRNA2). (B) Western blot analyses the expression of TLR2, CD133, Oct4, Sox2, C-myc, Nanog, p-YAP, YAP and HIF-1α in CD133^-^ PaTu8988 cancer cells or TLR2 silenced(TLR2 shRNA2) CD133^-^ cancer cells treated with rhHMGB1(250ng/ml) for the indicated time. (C)Western blot analyses the expression of TLR2, CD133, Sox2, and Nanog in CD133^-^ cancer cells or YAP silenced (YAP shRNA2) CD133^-^ cancer cells treated with rhHMGB1(250ng/ml) for the indicated time. (D)Western blot analyses of shRNA knockdown efficiency of YAP protein in CD133^-^PaTu8988. (E) Western blot analyses the expression of YAP in the nuclear of HIF-1α silenced CD133^-^ PaTu8988 cancer cells treated with or without rhHMGB1. Experiments were repeated three times and the data were expressed as mean±SEM. Student's t-test, Oneway, two-sided ANOVA. **P*<0.05, ***P*<0.01, ****P*<0.001.

**Figure S4** Quantitative analysis of the ratio of p-YAP (Ser397) to YAP at protein level in CD133^-^ cancer cells or sh-TLR2 silenced CD133^-^ cancer cells treated with rhHMGB1(250ng/ml) and with or without Stevioside. Experiments were repeated three times and the data were expressed as mean±SEM. Student's t-test, Oneway, two-sided ANOVA.

**Figure S5** (A) Pluripotent genes (Oct4, Sox4, C-myc, Nanog) promoter genomic DNA sequences for matching to the consensus HIF-1α and YAP binding-site sequence were identified. (C) ChIP assay accessed YAP band to site of pluripotent genes (Oct4, Sox4, C-myc, Nanog) promoter genomic DNA sequence treated with or without rhHMGB1 in CD133^-^ cancer cells and HIF-1α knockdown CD133^-^ cancer cells. Experiments were repeated three times and the data were expressed as mean±SEM. Student's t-test, Oneway, two-sided ANOVA. **P*<0.05, ***P*<0.01, ****P*<0.001.

**FigureS6** Role of Bio-HMGB1 (redox state and oxidized state) inducing the expression of stem cell related markers (CD133, Oct4, Sox2, c-myc, Nanog and HIF-1α) in CD133^-^ PaTu8988 cancer cells. Experiments were repeated three times and the data were expressed as mean±SEM. Student's t-test, Oneway, two-sided ANOVA. **P*<0.05, ***P*<0.01, ****P*<0.001.

**Table 1. Sequences of shRNAs**

| Name | Sequence |
| --- | --- |
| HMGB1-shRNA1 | 5’-CCGGCCCAGATGCTTCAGTCAACTTCTCGAGAAGTTGACTGAAGCATCTGGGTTTTTG-3’ |
| HMGB1-shRNA2 | 5’-CCGGCCGTTATGAAAGAGAAATGAACTCGAGTTCATTTCTCTTTCATAACGGTTTTT-3’ |
| TLR2-shRNA1 | 5’-CCGGGCATCTGATAATGACAGAGTTCTCGAGAACTCTGTCATTATCAGATGCTTTTTG-3’ |
| TLR2-shRNA2 | 5’-CCGGGCACACGAATACACAGTGTAACTCGAGTTACACTGTGTATTCGTGTGCTTTTTG-3’ |
| YAP-shRNA1 | 5’-CCGGCCCAGTTAAATGTTCACCAATCTCGAGATTGGTGAACATTTAACTGGGTTTTTG-3’ |
| YAP-shRNA2 | 5‘ -CCGGGCCACCAAGCTAGATAAAGAACTCGA GTTCTTTATCTAGCTTGGTGGCTTTTTG-3’ |
| HIF-1α-shRNA1 | 5’-CCGGGTGATGAAAGAATTACCGAATCTCGAGATTCGGTAATTCTTTCATCACTTTTT-3’ |
| HIF-1α-shRNA2 | 5’-CCGGTGCTCTTTGTGGTTGGATCTACTCGAGTAGATCCAACCACAAAGAGCATTTTT-3’ |

**Table 2. Sequences of primers used for qRT-PCR**

| Name | Direction | Sequence (5’-3’) |
| --- | --- | --- |
| Oct4 | Forward | 5’-AAAGCAGAAACCCTCGT-3’ |
|  | Reverse | 5’-TCCAGGTTGCCTCTCAC-3’ |
| Sox2 | Forward | 5’- CCCCTGGCATGGCTCTTGGC -3’ |
|  | Reverse | 5’- TCGGCGCCGGGGAGATACAT -3’ |
| Nanog | Forward | 5’-GAGACAGAAATACCTCAGCC-3’ |
|  | Reverse | 5’-TCTGCGTCACACCATTG-3’ |
| c-myc | Forward | 5’-GACCAGCTGGAGATGGTGAC-3’ |
|  | Reverse | 5’-GGTCGCAGATGAAACTCTGG-3’ |
| cyclinE | Forward | 5’-ATCCCCACACCTGACAAAGAAG-3’ |
|  | Reverse | 5’-CCTGAACAAGCTCCATCTGTCA-3’ |
| SPP1 | Forward | 5’-GGAUGAUAUGGAUGAUGAAGA-3’ |
|  | Reverse | 5’-GAACGACUCUGAUGAUGUAGA-3’ |
| CTGF | Forward | 5’-GCAGGCTAGAGAAGCAGAGC-3’ |
|  | Reverse | 5’-ATGTCTTCATGCTGGTGCAG-3’ |
| HIF-1a | Forward | 5’-CA CCACAGGACAGTACAGGAT-3’ |
|  | Reverse | 5’-CGTGCTGAATAATACCACTCACA-3’ |

**Table 3. Sequences of primers used for HRE Site**

| Name | Direction | Sequence (5’-3’) |
| --- | --- | --- |
| Oct4 | Forward | AGGAGGCAAGTGAGCTTCGAC |
|  | Reverse | AGTTGATACACACCCCTCCCTG |
| Sox2 | Forward | GGGAGATGGCAGCTTACCAA |
|  | Reverse | AGGTGCCAACACTCTCTCAC |
| Nanog | Forward | TGGGAGGAGGGATAGACAAGA |
|  | Reverse | AGTGTCTCAAAATCTGGCTAGT |
| C-myc | Forward | CCCCCAACAAATGCAATGGG |
|  | Reverse | CCCAGGGAGAGTGGAGGAAA |

**Table 4. Sequences of primers used for TEAD Site**

| Name | Direction | Sequence (5’-3’) |
| --- | --- | --- |
| Oct4 | Forward | TATAGAGCTGCTGCGGGATTG |
|  | Reverse | ACTCCAGAAACCAGTCACACC |
| Sox2 | Forward | GAGATGTGGCTGGGGCTAAG |
|  | Reverse | GCTCGGACCATAAGGCAGAC |
| Nanog | Forward | TTCAGGTTCTGTTGCTCGGT |
|  | Reverse | TCCCGTCTACCAGTCTCACC |
| C-myc | Forward | TGCTTTGGCAGCAAATTGGG |
|  | Reverse | ACTTGGAGAGCGCGTTATGA |
